# Pre-Existing Heart Failure, Biomarker Profiles, and Patients’ Vulnerability in Hospitalized COVID-19: A Biomarker-Driven Risk Framework

**DOI:** 10.3390/jcm15051909

**Published:** 2026-03-03

**Authors:** Ana-Maria Pah, Maria-Laura Craciun, Adina Avram, Ruxandra Maria Christodorescu, Daian Ionel Popa, Simina Crisan, Cristina Vacarescu, Diana-Maria Mateescu, Dragos-Mihai Gavrilescu, Florina Buleu, Adrian-Cosmin Ilie

**Affiliations:** 1 Cardiology Department, “Victor Babes” University of Medicine and Pharmacy, Eftimie Murgu Square 2, 300041 Timisoara, Romania; anamaria.pah@umft.ro (A.-M.P.); laura.craciun@umft.ro (M.-L.C.); simina.crisan@umft.ro (S.C.); cristina.vacarescu@umft.ro (C.V.); 2 Department of Internal Medicine I, “Victor Babes” University of Medicine and Pharmacy, Eftimie Murgu Square 2, 300041 Timisoara, Romania; 3 Research Center for Medical Communication, “Victor Babes” University of Medicine and Pharmacy, Eftimie Murgu Square 2, 300041 Timisoara, Romania; daian-ionel.popa@umft.ro; 4 Doctoral School, Department of General Medicine, “Victor Babes” University of Medicine and Pharmacy, Eftimie Murgu Square 2, 300041 Timisoara, Romania; diana.mateescu@umft.ro; 5 Department of Orthodontics, Dental District, Strada Zăgazului Nr. 3, One Floreasca Vista, Sector 1, 014261 Bucharest, Romania; dr.gavrilescu@outlook.com; 6 Department VI, Discipline of Internal Medicine and Ambulatory Care, Prevention and Cardiovascular Recovery, Faculty of Medicine, “Victor Babes” University of Medicine and Pharmacy, Eftimie Murgu Square 2, 300041 Timisoara, Romania; florina.buleu@umft.ro; 7 Department of Public Health and Sanitary Management, “Victor Babes” University of Medicine and Pharmacy, Eftimie Murgu Square 2, 300041 Timisoara, Romania; ilie.adrian@umft.ro; 8 Centre for Translational Research and Systems Medicine, Faculty of Medicine, “Victor Babeș” University of Medicine and Pharmacy, Eftimie Murgu Square No. 2, 300041 Timișoara, Romania

**Keywords:** COVID-19, heart failure, sepsis, mortality, cardiac biomarkers, NT-proBNP, high-sensitivity troponin, inflammatory biomarkers

## Abstract

**Background/Objectives**: Heart failure (HF) has been associated with adverse outcomes in coronavirus disease 2019 (COVID-19), but it remains unclear whether HF independently predicts sepsis and mortality once inflammatory and cardiac biomarkers are considered. **Methods**: This single-center retrospective cohort analysis included 127 adult patients hospitalized with laboratory-confirmed COVID-19 at a tertiary infectious diseases hospital between March 2020 and December 2024. Pre-existing HF was defined based on cardiology records and chronic HF therapy. Baseline assessments included clinical characteristics, echocardiography, and biomarkers (NT-proBNP, high-sensitivity troponin, C-reactive protein [CRP], interleukin 6 [IL-6], procalcitonin, and D-dimer) measured within 24 h of admission. Primary outcomes were sepsis and all-cause mortality (in-hospital or 30-day). Independent associations with sepsis and mortality were examined using multivariable logistic regression incorporating demographic factors, major comorbidities, baseline disease severity, and inflammatory and cardiac biomarkers. **Results**: Of 127 patients (mean age 70.1 ± 14.8 years, 63.8% male), 30 (23.6%) had pre-existing HF. Patients with preexisting heart failure exhibited significantly reduced left ventricular ejection fraction and higher admission levels of NT-proBNP and high-sensitivity troponin, accompanied by a substantially increased burden of in-hospital cardiovascular complications (53.3% vs. 14.4%, *p* < 0.001). However, sepsis (6.7% vs. 7.2%, *p* = 1.000) and total mortality (20.0% vs. 17.5%, *p* = 0.971) did not differ significantly between HF and non-HF groups. In multivariable analyses, HF was not independently associated with sepsis (adjusted odds ratio [aOR] 0.78, 95% confidence interval [CI] 0.05–12.34, *p* = 0.855) or mortality (aOR 0.63, 95% CI 0.16–2.46, *p* = 0.506). By contrast, higher CRP (aOR 1.01 per 1 mg/L, 95% CI 1.00–1.01, *p* = 0.007), IL-6 (aOR 1.01 per 1 pg/mL, 95% CI 1.00–1.01, *p* = 0.025), and high-sensitivity troponin (aOR >999, 95% CI 138–>999, *p* = 0.001) were independently associated with mortality. **Conclusions**: In hospitalized COVID-19 patients, pre-existing HF identifies a subgroup with heightened cardiac biomarker activation and a substantially higher burden of cardiovascular complications but does not associate with sepsis or short-term mortality in this cohort after adjustment for inflammatory and cardiac biomarkers. Mortality risk appears to be driven primarily by systemic inflammation and acute myocardial injury, as reflected by CRP, IL-6, and high-sensitivity troponin. These findings support a biomarker-driven approach to risk stratification in COVID-19, in which dynamic inflammatory and cardiac injury profiles provide more prognostic information than HF status alone, while still warranting intensified cardiovascular surveillance in patients with HF.

## 1. Introduction

COVID-19 emerged as a clinical syndrome following the spread of the novel coronavirus SARS-CoV-2, producing a wide range of respiratory and systemic manifestations, with highly variable clinical presentations ranging from self-limited upper respiratory illness to life-threatening pneumonia, systemic inflammatory response, sepsis, and multiorgan failure [1,2]. Large observational cohorts further demonstrated that older age and comorbidity burden—particularly cardiovascular disease—are strongly associated with adverse outcomes and in-hospital mortality among hospitalized patients [3]. These observations emphasize clinical vulnerability as a central determinant of prognosis during SARS-CoV-2 infection.

Myocardial injury is a frequent complication of COVID-19 and has been consistently linked to disease severity, intensive care unit admission, and mortality. Studies reporting elevated cardiac troponin identified a strong association between myocardial injury and death in hospitalized patients, supporting the prognostic relevance of cardiac involvement in COVID-19 [4,5]. In bigger cohorts, myocardial injury remained prevalent and clinically meaningful, reinforcing that cardiac injury is not an incidental finding but a key contributor to adverse outcomes [6].

Heart failure represents a particularly high-risk clinical phenotype in the context of COVID-19. Beyond being a marker of frailty and advanced cardiovascular disease, heart failure may amplify susceptibility to hypoxia, hemodynamic instability, and inflammatory injury, thereby predisposing patients to complications during acute infection. Contemporary reviews have summarized multiple pathophysiological pathways linking COVID-19 to myocardial dysfunction and decompensated heart failure [7]. Observational evidence further suggests that heart failure in COVID-19 is associated with unfavorable clinical trajectories and higher mortality compared with patients without heart failure [8,9].

Cardiac biomarkers provide additional prognostic and mechanistic insights. N-terminal pro-B-type natriuretic peptide (NT-proBNP), reflecting myocardial wall stress and ventricular dysfunction, is frequently elevated in hospitalized COVID-19 patients and has been associated with mortality risk, including in severe disease [10]. Troponin elevation likewise represents the extent of myocardial injury and correlates with outcomes, complementing the clinical characterization of cardiac involvement in COVID-19 [4,5,6].

Systemic inflammation and thromboinflammation are pivotal drivers of disease progression in COVID-19. Classic early clinical cohorts demonstrated that severe disease is accompanied by marked inflammatory activation and organ dysfunction, providing a biological basis for sepsis and multiorgan failure in hospitalized patients [1,3]. Biomarker-based analyses identified inflammatory signals that predict mortality, supporting the relevance of immune dysfunction in critical illness and sepsis evolution [11]. In parallel, comprehensive cardiovascular-focused reviews summarized how SARS-CoV-2–related inflammation and endothelial dysfunction intersect with cardiovascular disease, worsening outcomes [12]. Hypercoagulability and thrombotic complications add further to organ injury and hemodynamic deterioration, with significant consequences for prevention and management in hospitalized patients [13].

Although numerous studies have evaluated either heart failure or individual biomarkers as prognostic markers in COVID-19, fewer have assessed whether heart failure remains an independent predictor of sepsis and mortality within an integrated framework that includes both cardiac and inflammatory biomarkers. Moreover, clinically overt myocarditis—albeit less common—illustrates the plausibility of direct inflammatory myocardial involvement causing acute ventricular dysfunction and decompensation [14].

Therefore, the present study aimed to determine whether pre-existing heart failure independently predicts sepsis and mortality in hospitalized COVID-19 patients and to elucidate the contributory roles of inflammatory and cardiac biomarkers in these associations.

## 2. Materials and Methods

### 2.1. Study Design and Setting

This retrospective cohort study was conducted at the “Dr. Victor Babeș” Clinical Hospital of Infectious Diseases and Pneumophthisiology, a tertiary referral center in Timișoara, Romania. All patients were admitted to the Department of Infectious Diseases I for management of confirmed coronavirus disease 2019 (COVID-19). Consecutive adults hospitalized between March 2020 and December 2024 were evaluated for eligibility.

### 2.2. Study Population

COVID-19 was diagnosed in accordance with national and institutional protocols, based on a positive reverse transcription polymerase chain reaction (RT PCR) assay or a validated antigen test detecting severe acute respiratory syndrome coronavirus 2 (SARS-CoV-2). The cohort comprised hospitalized adults with confirmed COVID-19 and early cardiac and inflammatory biomarker assessment.

During the study period, a total of 245 consecutive adults were hospitalized with confirmed COVID-19 in our department. Of these, 118 patients were excluded due to missing core biomarker data (*n* = 74), incomplete information on pre-existing heart failure or major comorbidities (*n* = 19), missing outcome data for sepsis or mortality (*n* = 11), transfer from another hospital after a prolonged prior admission (*n* = 9), or admissions in which acute SARS-CoV-2 infection was not the primary clinical problem (*n* = 5). The final analysis included 127 patients with complete data for the predefined variables of interest.

We excluded patients with incomplete information on pre-existing heart failure or other major comorbidities, missing core biomarker data (NT-proBNP, troponin, CRP, IL-6, procalcitonin, D-dimer), or missing outcome data (sepsis or mortality). Additional exclusion criteria were transfer from another hospital after a prolonged prior admission and admissions in which acute SARS-CoV-2 infection was not the primary clinical problem.

### 2.3. Data Collection

Electronic hospital records were reviewed for clinical and laboratory information. Baseline variables included demographics, BMI, vaccination status, symptom timing, and admission vital signs. Comorbidity data included a history of heart failure, ischemic heart disease, hypertension, diabetes mellitus, chronic kidney disease, chronic liver disease, chronic lung disease, obesity, and prior thromboembolic events.

Disease severity at presentation was classified as mild, moderate, severe, or critical, based on respiratory impairment, oxygen requirement, and radiologic findings, according to contemporaneous national and World Health Organization guidance. Lung involvement was classified as absent, unilateral, or bilateral on chest imaging.

The in-hospital course was documented, including the need for supplemental oxygen and its modality, intensive care unit (ICU) admission, use and duration of invasive mechanical ventilation, development of sepsis or other complications (renal or cardiovascular), length of hospital stay, and vital status at discharge.

Survivors were followed for up to 3 months after hospital discharge when data were available through outpatient visits or medical record review. Follow-up assessments included persistent respiratory symptoms, New York Heart Association (NYHA) functional class, resting and exertional oxygen saturation, 6-min walk distance, follow-up NT-proBNP and troponin levels, left ventricular ejection fraction, and radiologic evidence of residual pulmonary fibrosis. Among the 104 survivors, follow-up data at 3 months were available for 83 patients (79.8%).

### 2.4. Biomarker Assessment

All laboratory analyses were performed in the central accredited laboratory of the “Dr. Victor Babeș” Clinical Hospital of Infectious Diseases and Pneumophthisiology. Blood cell counts were determined using automated laboratory systems (Sysmex XN 1000, Sysmex Corporation, Kobe, Japan).

C-reactive protein levels were assessed using immunoturbidimetric methods on a Cobas Integra 400 Plus analyzer (Roche Diagnostics, Mannheim, Germany), and interleukin 6 (IL-6) concentrations were determined using an electrochemiluminescence immunoassay (Elecsys IL-6, Cobas e601, Roche Diagnostics, Mannheim, Germany). D-dimer levels were quantified using an immunoturbidimetric method (STA Liatest D Di, Diagnostica Stago, Asnières sur Seine, France) on the STA Compact Max analyzer (Diagnostica Stago, France). Procalcitonin was assessed by a chemiluminescent immunoassay according to manufacturer protocols.

Cardiac biomarkers included N-terminal pro-B-type natriuretic peptide (NT-proBNP), reflecting myocardial wall stress, and high-sensitivity cardiac troponin, reflecting myocardial injury; both were measured using automated immunoassays in compliance with manufacturer instructions and internal quality control procedures. Serum creatinine, urea, liver enzymes (aspartate aminotransferase, alanine aminotransferase), albumin, and serum lactate were also recorded, together with arterial blood gas parameters (PaO_2_, PaCO_2_, pH, bicarbonate, SaO_2_/FiO_2_ ratio).

### 2.5. Echocardiography and Definition of Heart Failure

Transthoracic echocardiography was performed or reviewed by experienced cardiologists using standardized protocols. Left ventricular ejection fraction (LVEF) was assessed by the biplane Simpson method, and diastolic dysfunction was graded according to contemporary echocardiographic guidelines.

Heart failure was defined as a pre-existing clinical diagnosis established before the index admission, irrespective of LVEF phenotype (reduced, mildly reduced, or preserved), based on cardiology records and chronic heart failure treatment. For descriptive purposes, HF phenotypes were categorized as heart failure with reduced ejection fraction (HFrEF, LVEF <40%), mildly reduced ejection fraction (HFmrEF, LVEF 40–49%), and preserved ejection fraction (HFpEF, LVEF ≥50%), according to contemporary guidelines. Patients with a documented history of heart failure hospitalization or ongoing guideline-directed medical therapy for heart failure were classified as having heart failure. When available, baseline NYHA class and echocardiographic parameters (LVEF and diastolic function) were abstracted.

### 2.6. Definitions of Outcomes

The primary outcomes were sepsis and mortality. Sepsis was defined according to Sepsis-3 criteria as suspected or confirmed infection associated with acute organ dysfunction. In the context of SARS-CoV-2 infection, sepsis was operationalized as new or worsening organ dysfunction during hospitalization, reflected by clinical deterioration requiring escalation of respiratory or hemodynamic support together with laboratory evidence of organ injury (e.g., acute kidney injury, hepatic dysfunction, elevated lactate) or an increase in Sequential Organ Failure Assessment (SOFA) score of ≥2 points when available. Sepsis was ascertained through retrospective chart review, requiring documentation of infection-related organ dysfunction (e.g., hypotension requiring vasopressors, new or worsening respiratory failure, acute kidney injury, or need for high-flow oxygen or mechanical ventilation) together with a compatible clinical picture. Diagnoses were established and verified by the treating infectious disease physicians in accordance with institutional protocols.

Mortality endpoints included all-cause in-hospital death and 30-day mortality.

Secondary outcomes comprised ICU admission, need and duration of invasive mechanical ventilation, acute kidney injury, and in-hospital cardiovascular complications, defined as acute decompensated heart failure, clinically significant arrhythmias (including new-onset or worsening atrial fibrillation), and acute coronary syndromes.

### 2.7. Statistical Analysis

Continuous variables are summarized using measures of central tendency and dispersion, and categorical variables as proportions. Group differences were assessed using appropriate parametric or nonparametric tests according to data distribution.

Due to their right-skewed distribution, inflammatory biomarkers (CRP, IL-6, procalcitonin, and D-dimer) are presented as median (IQR) and were log-transformed where appropriate for regression analyses. Univariable logistic regression was initially used to evaluate crude associations between heart failure, individual biomarkers, and the primary outcomes (sepsis and mortality).

Multivariable logistic regression was used to assess independent associations between heart failure, inflammatory and cardiac biomarkers, and clinical outcomes, including sepsis and mortality. Models were adjusted for clinically essential covariates (age, sex, and admission COVID-19 severity) together with selected biomarkers chosen based on clinical relevance while avoiding redundancy among correlated inflammatory markers. To improve interpretability and reduce the influence of extreme values, biomarkers were rescaled where appropriate (e.g., CRP per 10 mg/L and high-sensitivity troponin per 0.01 ng/mL increase). Given the skewed distribution and the extreme odds ratios observed in exploratory models, high-sensitivity troponin was analyzed using a rescaled linear term (per 0.01 ng/mL) rather than a log-transformed unit, to facilitate clinical interpretation while avoiding implausible OR estimates.

Given potential temporal heterogeneity related to evolving SARS-CoV-2 variants, vaccination coverage, and treatment protocols, we conducted exploratory sensitivity analyses, additionally adjusting for admission period (2020–2021 vs. 2022–2024) and vaccination status. These covariates were not retained in the parsimonious mortality model to avoid overfitting, but were used to assess the robustness of the main associations.

Given the modest sample size and the low number of outcome events, model complexity was intentionally limited to reduce the risk of overfitting. Multivariable models were prespecified to include a small set of clinically essential covariates followed by selected biomarkers. Model performance was evaluated by reporting odds ratios (ORs) with 95% confidence intervals (CIs), while discrimination and calibration were explored using standard diagnostic measures.

Internal validation was performed using bootstrap resampling with 200 repetitions to estimate optimism-corrected model performance and to quantify potential overfitting through shrinkage.

Analyses were conducted using a complete-case approach. Patients with missing core biomarker or outcome data were excluded during cohort construction, and no imputation of missing values was performed. For variables with sporadic missingness among included patients, multivariable models were estimated using listwise deletion.

All analyses were conducted using IBM SPSS Statistics for Windows, version 26.0 (IBM Corp., Armonk, NY, USA), with statistical significance set at *p* < 0.05.

### 2.8. Ethical Considerations

Ethical principles and national regulatory requirements were observed throughout the study. The research protocol was approved by the Ethics Committee of the “Dr. Victor Babeș” Hospital of Infectious Diseases and Pneumophthisiology, Timișoara, Romania (approval no. 11901/16.12.2025). Given the retrospective design and use of anonymized data, the requirement for additional study-specific informed consent was waived. At hospital admission, all patients had provided written informed consent for hospitalization and for the potential use of anonymized clinical data for research purposes, according to institutional policy.

## 3. Results

### 3.1. Baseline Characteristics of the Study Population

A total of 127 hospitalized COVID-19 patients were included in the analysis, with a mean age of 70.1 ± 14.8 years and 63.8% male. Preexisting heart failure (HF) was present in 30 patients (23.6%), while 97 patients (76.4%) had no prior HF diagnosis. Patients with HF were similar in age to those without HF (mean 68.0 ± 14.2 years vs. 70.6 ± 15.0 years, *p* = 0.350) but exhibited lower left ventricular ejection fraction (LVEF) at baseline echocardiography (median 39% [IQR 35–45%] vs. 58% [IQR 54–62%], *p* < 0.001). Diastolic dysfunction was more prevalent and severe in the HF group (*p* = 0.002), with 40% graded as mild (grade 1) compared to 39% in non-HF patients. Among patients with pre-existing heart failure, 18 (60%) had HFrEF (LVEF <40%), 6 (20%) had HFmrEF (LVEF 40–49%), and 6 (20%) had HFpEF (LVEF ≥50%), based on the most recent available echocardiogram prior to admission. The distribution of HF phenotypes is summarized in Table 1.

Comorbidity profiles showed no significant differences in hypertension (50% in HF vs. 59% in non-HF, *p* = 0.525), diabetes (33% vs. 28%, *p* = 0.727), or obesity (27% vs. 33%, *p* = 0.670). However, patients with HF were more likely to receive diuretics (90% vs. 19%, *p* < 0.001) and antibiotics (73% vs. 47%, *p* = 0.023). COVID-19 severity at admission was comparable between groups (*p* = 0.462), with moderate disease being the most common (77% in HF vs. 61% in non-HF). Bilateral pulmonary infiltrates were observed in 70% of HF patients and 74% of non-HF patients (*p* = 0.891). Atrial fibrillation was more common in patients with pre-existing HF than in those without HF (36.7% vs. 9.3%, *p* < 0.001), consistent with the expected comorbidity burden in this population.

Vital signs and laboratory parameters at admission are detailed in Table 1. Notably, HF patients had higher D-dimer levels (median 1074 ng/mL [IQR 638–2035] vs. 740 ng/mL [IQR 414–1315], *p* = 0.017) but lower IL-6 (median 23 pg/mL [IQR 12–64] vs. 53 pg/mL [IQR 22–92], *p* = 0.043). Leukocyte counts were lower in HF (median 7.1 × 10^9^/L vs. 8.9 × 10^9^/L, *p* = 0.048), and arterial pH was higher (median 7.42 vs. 7.39, *p* = 0.017). No differences were observed in CRP, procalcitonin, or oxygenation parameters (SaO_2_/FiO_2_ ratio: median 239 vs. 201, *p* = 0.128).

### 3.2. Inflammatory and Cardiac Biomarkers

**Figure 1:**
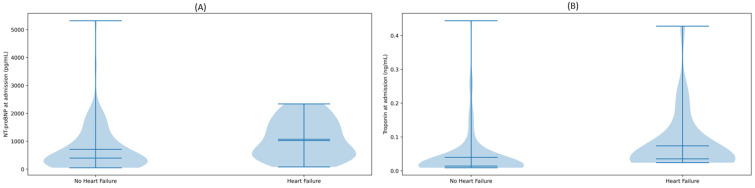
Admission levels of N-terminal pro-B-type natriuretic peptide (NT-proBNP) and high-sensitivity troponin according to pre-existing heart failure status in hospitalized COVID-19 patients. (**A**) NT-proBNP levels in patients with and without heart failure. (**B**) High-sensitivity troponin levels in patients with and without heart failure. Both biomarkers showed higher levels in patients with heart failure compared with those without (NT-proBNP *p* = 0.002, high-sensitivity troponin *p* = 0.001). The embedded boxplots indicate the median (central line), interquartile range (box), and full data range (whiskers).

Cardiac biomarkers at admission were significantly higher in patients with pre-existing HF compared with those without HF (Table 2). Median NT-proBNP was 1023 pg/mL [IQR 475–1648] in the HF group versus 396 pg/mL [IQR 169–925] in the non-HF group (*p* = 0.002), indicating greater myocardial wall stress. High-sensitivity troponin levels were likewise higher in HF patients (median 0.036 ng/mL [IQR 0.031–0.083] vs. 0.014 ng/mL [IQR 0.014–0.029], *p* < 0.001), consistent with more pronounced myocardial injury. Admission cardiac biomarkers thus differed substantially by HF status; as illustrated in Figure 1, NT-proBNP and high-sensitivity troponin values were markedly higher in patients with pre-existing HF than in those without HF (*p* = 0.002 and *p* < 0.001, respectively).

Inflammatory biomarkers showed heterogeneous patterns according to HF status (Table 2). D-dimer was significantly elevated in HF patients compared with non-HF patients (median 1074 ng/mL [IQR 638–2035] vs. 740 ng/mL [IQR 414–1315], *p* = 0.017), whereas IL-6 levels were lower in the HF group (median 23 pg/mL [IQR 12–64] vs. 53 pg/mL [IQR 22–92], *p* = 0.043). CRP and procalcitonin did not differ significantly between groups (CRP: *p* = 0.725; procalcitonin: *p* = 0.164), suggesting that not all inflammatory pathways are uniformly modulated by HF status in this cohort.

### 3.3. Clinical Outcomes

In-hospital sepsis occurred in 7% of HF patients and 7% of non-HF patients (*p* = 1.000). All-cause mortality (in-hospital or 30-day) was 20% in HF vs. 18% in non-HF (*p* = 0.971). ICU admission was required in 27% of HF patients vs. 18% of non-HF (*p* = 0.402), with mechanical ventilation in 10% vs. 11% (*p* = 1.000). Acute kidney injury was less common in HF (10% vs. 29%, *p* = 0.063), while cardiovascular complications were markedly higher (53% vs. 14%, *p* < 0.001). In-hospital cardiovascular complications (acute decompensated HF, clinically significant arrhythmias, or acute coronary syndromes) were markedly more frequent in patients with HF than in those without HF (53.3% vs. 14.4%, *p* < 0.001).

Hospital stay duration was similar (median 12.5 days in HF vs. 12 days in non-HF, *p* = 0.436). At 3-month follow-up (available for 80% of survivors), persistent dyspnea was reported in 20% of HF vs. 32% of non-HF (*p* = 0.303), with no differences in NYHA class or 6-min walk distance. Residual pulmonary fibrosis on CT was present in 13% of HF vs. 11% of non-HF (*p* = 1.000). Key clinical outcomes stratified by heart failure status are presented in Table 3.

### 3.4. Multivariable Associations of Heart Failure, Biomarkers, and Outcomes

In univariable analyses, pre-existing HF was not significantly associated with either sepsis (crude OR 0.92, 95% CI 0.18–4.71, *p* = 0.918) or all-cause mortality (crude OR 1.17, 95% CI 0.42–3.28, *p* = 0.766). By contrast, higher levels of inflammatory and cardiac biomarkers, particularly CRP, IL-6, D-dimer, and troponin, were associated with increased odds of both sepsis and death in crude models.

In multivariable logistic regression adjusted for age, sex, admission COVID-19 severity, heart failure status, CRP, and high-sensitivity troponin, heart failure was not independently associated with mortality (adjusted OR 0.63, 95% CI 0.09–4.23, *p* = 0.637). Higher admission severity (adjusted OR 2.45, 95% CI 1.66–3.61, *p* < 0.001) and high-sensitivity troponin (adjusted OR 1.15 per 0.01 ng/mL increase, 95% CI 1.04–1.27, *p* = 0.006) remained independently associated with death, whereas CRP did not retain independent significance after adjustment. In contrast to exploratory models yielding implausibly large odds ratios for hs-troponin, the parsimonious model with rescaled troponin (per 0.01 ng/mL) provided more stable and clinically interpretable estimates. After bootstrap internal validation, the optimism-corrected c-statistic for the mortality model was 0.95, with a calibration slope of 1.00, indicating good discrimination and calibration with minimal overfitting. The results of the parsimonious multivariable model are presented in Table 4.

In sensitivity analyses including admission period and vaccination status as additional covariates, the direction and magnitude of associations between heart failure, biomarkers, and mortality were generally similar, although confidence intervals widened and some predictors lost conventional statistical significance, consistent with the limited number of events.

**Figure 2:**
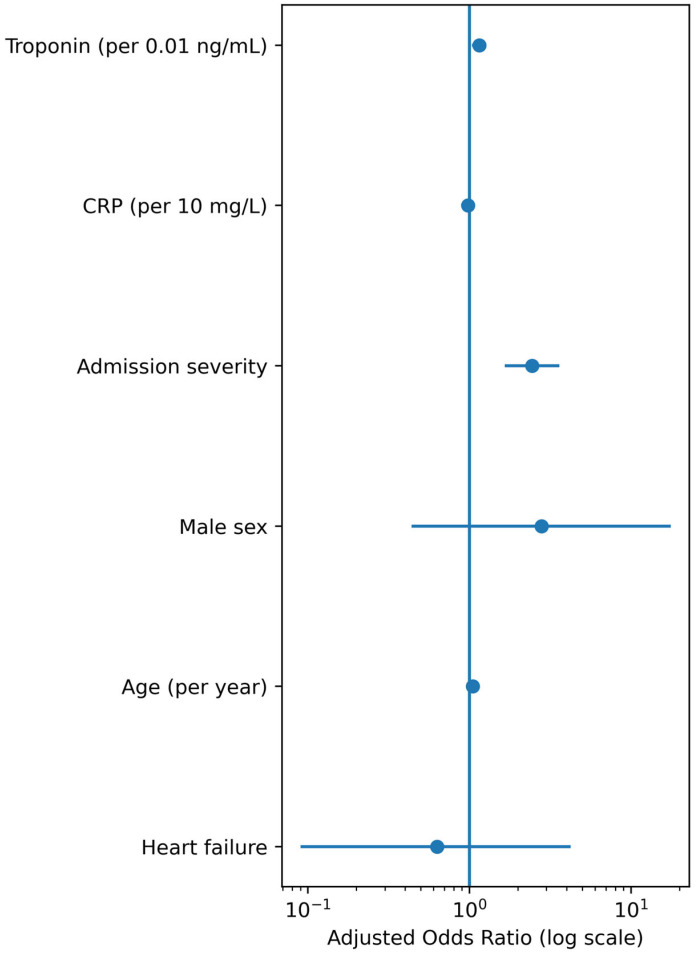
Forest plot showing adjusted odds ratios (ORs) and 95% confidence intervals (CIs) for all-cause mortality derived from the parsimonious multivariable logistic regression model including age, sex, admission COVID-19 severity, heart failure status, C-reactive protein (per 10 mg/L), and high-sensitivity troponin (per 0.01 ng/mL). Admission severity and troponin remained independently associated with mortality, whereas heart failure did not show an independent association with mortality in this model.

As summarized in Figure 2, these findings highlight that inflammatory and cardiac biomarkers, rather than HF status per se, drive mortality risk in this cohort.

Exploratory multivariable models for secondary outcomes showed that HF was strongly associated with in-hospital cardiovascular complications (adjusted OR 10.92, 95% CI 3.41–35.00, *p* < 0.001) and showed a trend toward a higher likelihood of ICU admission (adjusted OR 3.79, 95% CI 0.92–15.66, *p* = 0.065). Conversely, HF appeared inversely associated with acute kidney injury (adjusted OR 0.09, 95% CI 0.02–0.48, *p* = 0.005), a finding that may reflect differences in baseline diuretic use and clinical management rather than a true protective effect. Overall model performance was acceptable, with areas under the receiver operating characteristic curve between 0.72 and 0.85 and satisfactory calibration (Hosmer–Lemeshow *p* > 0.05).

## 4. Discussion

### 4.1. Principal Findings

In this single-center cohort of hospitalized COVID-19 patients, pre-existing heart failure identified a subgroup with more pronounced cardiac biomarker activation and a higher burden of in-hospital cardiovascular complications, but it did not independently associate with sepsis or short-term mortality in this cohort after adjustment for inflammatory and cardiac biomarkers. This evidence suggests that myocardial vulnerability in COVID-19 is expressed predominantly through biomarker elevation and clinical cardiovascular events, rather than through a direct, independent effect of heart failure status on short-term survival. In contrast, higher levels of CRP, IL-6, and high-sensitivity troponin emerged as independent predictors of mortality, highlighting the central role of systemic inflammation and myocardial injury as proximate drivers of adverse outcomes in this population. Collectively, these findings refine the prognostic paradigm in COVID-19 by indicating that a biomarker-based approach may more effectively capture risk than heart failure history alone in patients with established cardiovascular vulnerability.

### 4.2. Heart Failure, Cardiovascular Vulnerability, and Outcomes

The observed lack of an independent association between heart failure and sepsis or mortality aligns only partially with early reports that described heart failure as a strong risk marker for severe COVID-19 and death [15,16]. While large registries and multicenter cohorts consistently showed higher crude mortality among patients with pre-existing heart failure [17,18], many of these analyses did not simultaneously account for detailed biomarker profiles, competing comorbidities, and acute illness severity. In addition, prior work from our group demonstrated that the combined burden of heart failure and arterial hypertension was associated with adverse outcomes in hospitalized COVID-19 patients, further supporting the importance of overall cardiovascular comorbidity load in this setting [19]. In the present study, heart failure patients exhibited markedly higher NT-proBNP and troponin levels at admission, reflecting structural and functional myocardial compromise [20,21], yet these differences translated into excess cardiovascular complications rather than excess all-cause mortality once inflammatory and cardiac biomarkers were incorporated into multivariable models. The substantially higher prevalence of atrial fibrillation among HF patients in our cohort further reflects the advanced structural cardiac disease burden typical of this population and may have contributed to the increased rate of cardiovascular complications observed during hospitalization. This pattern suggests that heart failure in COVID-19 may function primarily as a substrate that facilitates acute decompensation, arrhythmias, and ischemic events, while the ultimate transition to sepsis and death is more tightly governed by the intensity of systemic inflammation and myocardial injury.

Several pathophysiological mechanisms may explain why heart failure did not retain independent prognostic significance in fully adjusted models. Patients with chronic heart failure often receive guideline directed medical therapy, including renin–angiotensin–aldosterone system inhibitors, beta blockers, and diuretics, which might partially mitigate hemodynamic stress and pulmonary congestion during acute infection [22,23]. Moreover, the close inpatient monitoring and lower threshold for escalation of care in patients with known heart failure could attenuate excess mortality despite a higher incidence of cardiovascular complications. The apparent inverse association between heart failure and acute kidney injury, together with the high prevalence of chronic diuretic use, supports the hypothesis that baseline treatment patterns and fluid management strategies may modulate organ-specific outcomes in this subgroup [24,25,26,27].

### 4.3. Role of Inflammatory and Cardiac Biomarkers in Risk Stratification

The robust associations between CRP, IL-6, and high-sensitivity troponin and mortality in multivariable analyses reinforce the central role of systemic inflammation and myocardial injury in the trajectory of severe COVID-19, consistent with prior studies demonstrating the prognostic value of inflammatory and cardiac biomarkers in hospitalized patients [28,29,30,31,32]. In this cohort, both biomarkers remained independently associated with mortality after controlling for age, comorbidities, and heart failure status, suggesting that inflammatory burden captures incremental risk beyond classic clinical predictors. High-sensitivity troponin, which reflects acute myocardial injury from ischemic, inflammatory, or supply–demand mechanisms, showed a particularly strong association with mortality, in line with prior studies that identified myocardial injury as a key determinant of adverse outcomes in hospitalized COVID-19 [30,31].

From a clinical perspective, these data support an approach to risk stratification that emphasizes dynamic inflammatory and cardiac biomarker profiles. This strategy appears more informative than relying on static comorbidity labels such as heart failure alone. Admission measurement of CRP, IL-6, NT-proBNP, and troponin, combined with standard clinical variables, could allow timely recognition of patients at increased risk for clinical worsening, independent of prior heart failure diagnosis. In settings where high-level imaging or hemodynamic monitoring is limited, such biomarker-based algorithms may provide a pragmatic means to guide triage decisions, ICU referral, and closer surveillance [33,34]. Additionally, the strong prognostic signals from IL-6 and troponin raise the possibility that patients with marked inflammatory or myocardial injury profiles might derive particular benefit from targeted therapies, such as anti-cytokine agents or optimized cardioprotective strategies, though this remains to be tested in prospective interventional studies [35].

Our findings should also be interpreted in the context of prior work demonstrating a high prevalence of subclinical myocardial dysfunction and its prognostic relevance after COVID-19 pneumonia, including studies using speckle-tracking echocardiography to detect impaired left ventricular strain in patients without overt heart failure [36]. In that setting, subtle myocardial dysfunction may emerge as a sensitive marker of ongoing cardiac involvement and predict longer-term adverse outcomes, even when conventional measures of systolic function and clinical status remain preserved. By contrast, the present analysis focused on a relatively small cohort of hospitalized patients with clinically recognized, pre-existing heart failure and evaluated short-term sepsis and mortality during the index admission, within a multivariable framework that incorporated inflammatory and cardiac biomarkers. Within this biomarker-driven model, heart failure status itself did not retain independent prognostic significance once acute systemic inflammation and myocardial injury were accounted for, whereas high-sensitivity troponin and inflammatory markers remained strongly associated with short-term mortality. These observations suggest that subclinical myocardial dysfunction, overt heart failure, and biomarker-defined myocardial injury may capture partly distinct dimensions and time points of COVID-19-related cardiac vulnerability, and that their respective prognostic roles likely depend on disease phase, patient selection, and the specific outcomes under study.

### 4.4. Clinical Consequences and Future Directions

The dissociation between heart failure status and adjusted mortality risk has multiple practical consequences for the management of hospitalized COVID-19 patients with cardiovascular disease. First, clinicians should recognize that a label of heart failure, while important for forecasting decompensation and arrhythmic events, does not automatically confer excess sepsis or mortality risk once inflammatory and biomarker profiles are taken into account. Instead, careful interpretation of admission and early course CRP, IL-6, NT-proBNP, and troponin levels is vital to refine prognostic assessment and to prioritize monitoring intensity and therapeutic resources [37,38]. Second, the strong association between heart failure and cardiovascular complications accentuates the requirement for proactive hemodynamic optimization, judicious fluid and diuretic management, and alert surveillance for arrhythmias and ischemia in this high-risk subgroup, regardless of their overall sepsis or mortality risk.

### 4.5. Limitations

A key limitation is the modest sample size and, importantly, the low number of outcome events, which reduces statistical power and increases the risk of overfitting in multivariable models. This may yield unstable estimates and wide confidence intervals, and may contribute to extreme odds ratios observed for some predictors (e.g., hs-troponin) and for secondary outcomes such as AKI. Therefore, our adjusted estimates should be interpreted cautiously and primarily as exploratory, and we complemented model development with internal validation to quantify optimism and potential overfitting. Given the observational design and limited sample size, the identified associations should not be interpreted as causal relationships but rather as hypothesis-generating findings.

Furthermore, because of the limited sample size and low number of events among patients with heart failure, we were unable to perform robust subgroup analyses stratified by left ventricular ejection fraction phenotype (HFrEF, HFmrEF, HFpEF). Patients with very low ejection fraction might have experienced worse outcomes irrespective of the inflammatory response, but this hypothesis could not be formally tested in our cohort.

Another important limitation is temporal heterogeneity, as patients were enrolled between 2020 and 2024, spanning multiple waves with different predominant SARS-CoV-2 variants, vaccination uptake, and therapeutic protocols. These secular changes may have influenced baseline risk, biomarker profiles, and outcomes in ways that are only partially captured by our covariates. Although exploratory models adjusting for admission period and vaccination status did not materially change the overall pattern of associations, residual confounding by unmeasured temporal factors remains likely.

Follow-up data were not available for all patients, which may introduce selection bias in post-discharge outcome assessment.

Frailty and functional status were not systematically captured in our retrospective dataset; however, frailty is increasingly recognized as a major determinant of outcomes and risk stratification in patients with heart failure. Future studies (and, where feasible, secondary analyses) should incorporate frailty measures or pragmatic proxies (e.g., dependence status, mobility limitations, prior nursing care, or a frailty index derived from routinely collected deficits) to better disentangle heart failure phenotype from overall vulnerability.

These considerations are consistent with emerging data highlighting frailty as a key driver of adverse outcomes in heart failure populations and support its integration into future risk stratification frameworks [39].

## 5. Conclusions

In this retrospective cohort of hospitalized COVID-19 patients, pre-existing heart failure identified a subgroup with pronounced cardiac biomarker activation and a substantially higher burden of in-hospital cardiovascular complications. Still, it did not independently associate with sepsis or short-term mortality in this cohort after adjustment for inflammatory and cardiac biomarkers. These data suggest that clinical vulnerability in COVID-19 is expressed predominantly through acute myocardial injury and systemic inflammatory activation rather than through heart failure status alone.

Inflammatory markers, particularly C-reactive protein and interleukin-6, together with high-sensitivity troponin, were independently associated with mortality in adjusted analyses, highlighting the central role of dysregulated host immune response and myocardial injury as proximal drivers of adverse outcomes. By contrast, N-terminal pro-B-type natriuretic peptide and a prior diagnosis of heart failure reflected baseline myocardial stress and structural vulnerability yet did not provide incremental prognostic information once dynamic biomarker profiles were considered.

From a clinical perspective, these results support a biomarker-driven risk-stratification strategy for hospitalized COVID-19 patients, in which early assessment of inflammatory and cardiac injury markers provides more accurate prognostic discrimination than reliance on comorbidity labels alone. While patients with heart failure warrant intensified surveillance for cardiovascular complications and careful hemodynamic management, mortality risk appears to be governed primarily by the intensity of systemic inflammation and acute myocardial injury.

Future prospective multicenter studies incorporating serial biomarker measurements and stratification by heart failure phenotype are needed to confirm these observations and to integrate inflammatory and cardiac biomarkers into pragmatic risk prediction models. Such approaches may enable earlier identification of high-risk patients and facilitate the development of customized therapeutic strategies that target both immune dysregulation and myocardial injury in severe COVID-19. These findings may inform biomarker-guided clinical surveillance and early risk stratification strategies in hospitalized COVID-19 patients with cardiovascular disease.

## Figures and Tables

**Table 1:** Baseline characteristics of hospitalized COVID-19 patients stratified by preexisting heart failure (HF). Continuous variables are presented as mean ± SD or median [IQR] based on distribution; categorical variables as *n* (%). *p*-Values from *t*-test/Mann–Whitney U for continuous and chi-square/Fisher’s exact for categorical variables.

Variable	HF (*n* = 30)	Non-HF (*n* = 97)	*p*-Value
Demographics			
Age (years), mean ± SD	68.0 ± 14.2	70.6 ± 15.0	0.350
Male sex, *n* (%)	19 (63.3)	62 (63.9)	1.000
BMI (kg/m^2^), median [IQR]	26.4 [23.8–30.8]	27.3 [24.1–30.5]	0.406
Comorbidities, *n* (%)			
HFrEF (<40%)	18 (60.0)	0 (0)	<0.001
HFmrEF (40–49%)	6 (20.0)	0 (0)	<0.001
HFpEF (≥50%)	6 (20.0)	0 (0)	<0.001
Atrial fibrillation	11 (36.7)	9 (9.3)	<0.001
Hypertension	15 (50.0)	57 (58.8)	0.525
Ischemic heart disease	7 (23.3)	18 (18.6)	0.755
Diabetes mellitus	10 (33.3)	27 (27.8)	0.727
COPD/asthma	7 (23.3)	12 (12.4)	0.239
Obesity	8 (26.7)	32 (33.0)	0.670
Chronic kidney disease	0 (0.0)	14 (14.4)	0.061
COVID-19 Presentation			
Symptom duration (days), median (IQR)	5 (3–5)	4 (3–5)	0.567
Disease severity, *n* (%)			0.462
- Mild	2 (6.7)	13 (13.4)	
- Moderate	23 (76.7)	59 (60.8)	
- Severe	4 (13.3)	20 (20.6)	
- Critical	1 (3.3)	5 (5.2)	
Bilateral infiltrates, *n* (%)	21 (70.0)	72 (74.2)	0.891
Vital Signs, median [IQR]			
Systolic BP (mmHg)	132.5 (115–145)	123 (110–140)	0.177
Heart rate (bpm)	91 (82–102)	98 (85–110)	0.172
Respiratory rate (breaths/min)	22.5 (20–26)	24 (20–27)	0.452
SpO2 (%)	92.6 (90–95)	92 (89–95)	0.482
Treatment, *n* (%)			
Diuretics	27 (90.0)	18 (18.6)	<0.001
Antivirals	17 (56.7)	50 (51.5)	0.778
Corticosteroids	22 (73.3)	70 (72.2)	1.000
Anticoagulants	24 (80.0)	90 (92.8)	0.094
Antibiotics	22 (73.3)	46 (47.4)	0.023

Note: *p*-values were obtained using *t*-test or Mann–Whitney U tests for continuous variables and chi-square tests for categorical variables; Fisher’s exact test was applied for categorical variables with expected cell counts <5 (including HF phenotype categories).

**Table 2:** Inflammatory and cardiac biomarkers at admission stratified by heart failure status. *p*-Values from the Mann–Whitney U test.

Biomarker	HF (*n* = 30), Median [IQR]	Non-HF (*n* = 97), Median [IQR]	*p*-Value
NT-proBNP (pg/mL)	1023 (475–1648)	396 (169–925)	0.002
Troponin (ng/mL)	0.036 [0.031–0.083]	0.014 [0.014–0.029]	<0.001
CRP (mg/L)	84 (63–233)	89 (62–149)	0.725
IL-6 (pg/mL)	23 (12–64)	53 (22–92)	0.043
Procalcitonin (ng/mL)	0.29 [0.16–0.61]	0.44 [0.25–0.65]	0.164
D-dimer (ng/mL)	1074 (638–2035)	740 (414–1315)	0.017

**Table 3:** Key clinical outcomes stratified by heart failure status. *p*-Values from chi-square test or Mann–Whitney U test.

Outcome	HF (*n* = 30), *n* (%) or Median [IQR]	Non-HF (*n* = 97), *n* (%) or Median [IQR]	*p*-Value
Sepsis	2 (6.7)	7 (7.2)	1.000
Mortality (total)	6 (20.0)	17 (17.5)	0.971
ICU admission	8 (26.7)	17 (17.5)	0.402
Mechanical ventilation	3 (10.0)	11 (11.3)	1.000
Acute kidney injury	3 (10.0)	28 (28.9)	0.063
Cardiovascular complications	16 (53.3)	14 (14.4)	<0.001
Length of hospital stay (days)	12.5 (9–15)	12 (9–15)	0.436

**Table 4:** Parsimonious multivariable logistic regression model for all-cause mortality in hospitalized COVID-19 patients. Odds ratios are adjusted for age, sex, admission COVID-19 severity, heart failure status, C-reactive protein (per 10 mg/L), and high-sensitivity troponin (per 0.01 ng/mL).

Predictor	Adjusted OR	95% CI	*p*-Value
Age (per year)	1.05	0.98–1.12	0.156
Male sex	2.80	0.44–17.65	0.273
Admission COVID-19 severity	2.45	1.66–3.61	<0.001
Heart failure	0.63	0.09–4.23	0.637
CRP (per 10 mg/L)	0.98	0.89–1.07	0.640
High-sensitivity troponin (per 0.01 ng/mL)	1.15	1.04–1.27	0.006

## Data Availability

De-identified clinical, laboratory, and imaging data supporting the findings of this study are available from the corresponding authors upon reasonable request, subject to institutional data-sharing policies.

## Abbreviations

The following abbreviations are used in this manuscript:

AKI	Acute kidney injury
ARDS	Acute respiratory distress syndrome
BMI	Body mass index
BP	Blood pressure
CKD	Chronic kidney disease
COPD	Chronic obstructive pulmonary disease
CRP	C-reactive protein
CT	Computed tomography
COVID-19	Coronavirus disease 2019
D-dimer	Fibrin degradation product
HF	Heart failure
hs-Tn	High-sensitivity troponin
ICU	Intensive care unit
IL-6	Interleukin-6
IQR	Interquartile range
LVEF	Left ventricular ejection fraction
NT-proBNP	N-terminal pro-B-type natriuretic peptide
NYHA	New York Heart Association
OR	Odds ratio
RT-PCR	Reverse transcription polymerase chain reaction
SaO_2_	Arterial oxygen saturation
SARS-CoV-2	Severe acute respiratory syndrome coronavirus 2
SD	Standard deviation
